# Identification of Monoallelically Expressed Genes Associated with Economic Traits in Hanwoo (Korean Native Cattle)

**DOI:** 10.3390/ani12010084

**Published:** 2021-12-31

**Authors:** Kyu-Sang Lim, Hyung-Chul Kim, Bong-Hwan Choi, Ju-Whan Son, Kyung-Tai Lee, Tae-Jeong Choi, Yong-Min Cho, Han-Ha Chai, Jong-Eun Park, Woncheoul Park, Chiwoong Lim, Jun-Mo Kim, Dajeong Lim

**Affiliations:** 1 Department of Animal Science, Iowa State University, Ames, IA 50011, USA; kyusang0912@gmail.com; 2 Animal Genomics and Bioinformatics Division, National Institute of Animal Science, RDA, Wanju 55365, Jeollabuk-do, Korea; khc3365@naver.com (H.-C.K.); bhchoi@korea.kr (B.-H.C.); tdpro@naver.com (J.-W.S.); leekt@korea.kr (K.-T.L.); choi6695@korea.kr (T.-J.C.); variance@korea.kr (Y.-M.C.); hanha@korea.kr (H.-H.C.); jepark0105@korea.kr (J.-E.P.); wcpark1982@korea.kr (W.P.); 3 Department of Animal Science and Technology, Chung-Ang University, Anseong 17546, Gyeonggi-do, Korea; dlacldnd@cau.ac.kr

**Keywords:** Hanwoo, monoallelic expression, adult tissues, polymorphism, breeding value

## Abstract

**Simple Summary:**

Although most genes are expected to transcribe both alleles evenly in diploid animals, some genes show monoallelic expression (MAE). The expressed allele can be determined randomly or by parental origin (known as genomic imprinting). Here, we conducted genome-wide and transcriptome-wide screening of MAE genes in Korean cattle (Hanwoo) and identified tissue-specific MAE pattens. The effects of MAE genes on phenotypic variation were evaluated by association analysis with the breeding values of five traits that were used in a Hanwoo breeding program.

**Abstract:**

Hanwoo, an indigenous Korean cattle breed, has been genetically improved by selecting superior sires called Korean-proven bulls. However, cows still contribute half of the genetic stock of their offspring, and allelic-specific expressed genes have potential, as selective targets of cows, to enhance genetic gain. The aim of this study is to identify genes that have MAEs based on both the genome and transcriptome and to estimate their effects on breeding values (BVs) for economically important traits in Hanwoo. We generated resequencing data for the parents and RNA-sequencing data for the muscle, fat, and brain tissues of the offspring. A total of 3801 heterozygous single nucleotide polymorphisms (SNPs) in offspring were identified and they were located in 1569 genes. Only 14 genes showed MAE (seven expressing maternal alleles and seven expressing paternal alleles). Tissue-specific MAE was observed, and *LANCL1* showed maternal allele expression across all tissues. MAE genes were enriched for the biological process of cell death and angiogenesis, which included *ACKR3* and *PDCL3* genes, whose SNPs were significantly associated with BVs of lean meat production-related traits, such as weight at 12 months of age, carcass weight, and loin eye area. In the current study, monoallelically expressed genes were identified in various adult tissues and these genes were associated with genetic capacity in Hanwoo.

## 1. Introduction

Genomic imprinting is a unique epigenetic phenomenon in which certain genes have monoallelic expression (MAE) based on allelic parental origins [1]. That is, the maternal or paternal allele is suppressed during expression of the imprinted gene. Since the first discovery of genomic imprinting in mice in the 1980s, more than 100 imprinted genes have been reported in mice and humans [2] and are listed in the web-based imprinted gene database (http://www.geneimprint.com/, accessed on 1 October 2021). Furthermore, imprinting status varies in tissue- and developmental stage-specific ways [3].

For farm animals in selective breeding programs, these imprinted genes have important implications since the genetic merits of reciprocal heterozygotes at imprinted loci have different effects on phenotypes [4]. The effects of genomic imprinting on quantitative trait loci (QTL) for economical traits have been identified in cattle [5], pigs [6,7], and sheep [8]. In addition, the associations between polymorphisms of imprinted genes and quantitative traits have also been reported in livestock species. For example, *insulin-like growth factor 2* (*IGF2*) and *insulin-like growth factor 2 receptor* (*IGF2R*), which are expressed paternally and maternally, respectively, and were associated with growth traits and meat quality in cattle [9,10]. In cattle, Killian et al. first reported the imprinted gene *M6P*/*IGF2R* in cattle and suggested that the different imprinting status of *M6P*/*IGF2R* in species provided clues into divergent evolution [11]. Several studies also identified MAE of *Nesp55*, *XIST*, *IGF2*, *PEG3*, and *H19* [12,13,14]; these studies used a polymorphism-based approach for target candidate genes, which were chosen based on previous literature regarding genomic imprinting in other species. Next, genome-wide screening of MAE genes was applied for conceptus [15] and multiple adult tissues [16].

These studies implied that the imprinting genes and their causative SNPs could provide information on the genetic variations caused by genomic imprinting. The integration of imprinting parent-of-origin effects associated with trait(s) of interest in genomic selection has been proposed to predict future phenotypes in livestock species [17]. Therefore, the aims of this study were to screen MAE genes for three types of tissues in Hanwoo by genome and transcriptome-based approaches and to provide new insights into the relationship between SNPs of MAE genes and the breeding values of five traits that were implemented in the national genetic evaluation system of Hanwoo.

## 2. Materials and Methods

### 2.1. Experimental Animals and Sample Collection 

To identify MAE in Hanwoo, three animals from a family (father, mother, and offspring) were provided by the Hanwoo Experiment Station, National Institute of Animal Science, South Korea. The peripheral blood samples were collected for DNA extraction from all animals. For RNA-sequencing, tissue samples, such as muscle (the longissimus muscle and femoral muscle), fat (backfat and abdominal fat), and brain (pituitary gland and hypothalamus) were collected from the offspring after slaughtering at 37 months of age.

### 2.2. Extraction of DNA and RNA 

Genomic DNA was extracted from the peripheral blood using the QIAamp DNA Blood Maxi Kit (Qiagen, Gaithersburg, MD, USA) based on the manufacturer’s instructions. In addition, total RNA of offspring was extracted from the six tissues using TRIzol reagent (Life Technologies Co., Grand Island, NY, USA) based on the manufacturer’s instructions. DNA and RNA quantity and quality were confirmed using agarose gel electrophoresis and Nanodrop ND-1000 spectrophotometer (Nanodrop Technologies Inc., Wilmington, DE, USA).

### 2.3. Sequencing

We produced indexed shotgun paired-end libraries with approximately 500 bp inserts that were generated using TruSeq Nano DNA Library Prep Kit (Illumina, San Diego, CA, USA) following the standard Illumina sample-preparation protocol. Briefly, 200 ng of gDNA was fragmented by Covaris M220 (Woburn, MA, USA), resulting in a median fragment size of approximately 500 bp followed by end repair, A-tailing, and indexed adapter ligation (approximately 125 bp adapter). Then, gel-based size selection from 550 to 650 bp was conducted for the adapter-ligated DNA, and PCR amplification was performed for eight cycles for libraries. The size-selected libraries were analyzed by an Agilent 2100 Bioanalyzer (Agilent Technologies, Palo Alto, CA, USA) to determine the size distribution and check for adapter contamination. The resulting libraries were sequenced in an Illumina HiSeq 2500 (2 × 125-bp paired-end sequences) sequencer.

To construct cDNA libraries with the TruSeq RNA library kit (Illumina, San Diego, CA, USA), 1 μg of total RNA was used. The protocol consisted of polyA-selected RNA extraction, RNA fragmentation, random hexamer primed reverse transcription, and 100 nt paired-end sequencing by Illumina HiSeq 2000 (Illumina, San Diego, CA, USA). The libraries were quantified by qPCR according to the qPCR Quantification Protocol Guide and qualified using an Agilent Technologies 2100 Bioanalyzer (Agilent Technologies, Palo Alto CA, USA).

### 2.4. Identification of MAE Genes

The sequence reads were mapped to the Bos taurus reference genome (UMD 3.1.78) using Bowtie2 [18] and TopHat [19] with default settings for re-seq and RNA-seq, respectively. Variant calling was performed for resequencing data and RNA-sequencing data using the Genome Analysis Toolkit (GATK, version 3.6) HaplotypeCaller with standard settings [20]. The SNPs with QD < 2.0, FS > 60.0, MQ < 40.0, MQRankSum < −12.5, and ReadPosRankSum < −8.0 were filtered out. The SNPs located in the exon regions were screened based on the SNP annotation using SnpEff software [21] and only exonic SNPs with GQ ≥ 20 and DP ≥ 10 for re-seq data of sire and dam remained to get their genotypes correctly.

To access the parental origin of the expressed allele in the offspring, homozygote SNPs with opposite alleles from the parents were evaluated. Then, MAE SNPs were determined based on allelic expression patterns in the RNA-seq data of each tissue. For considering correct allele assignment, SNPs with GQ ≥ 20 and DP ≥ 10 from RNA-seq data were used.

### 2.5. Association Analysis and Functional Enrichment Analysis

A total of 203 Korean-proven bulls (KPN)s were used in the estimation of the effects of MAE SNPs on breeding values of five traits that are routinely evaluated in Hanwoo [22]. Genotype data and estimated breeding values (BVs), including weight at 12 months of age (WT12), carcass weight (CWT), loin eye area (LEA), backfat thickness (BFT), and marbling (MAR), for these animals were provided by the Hanwoo Improvement Center, National Agricultural Cooperative Federation, Korea. The association analysis was performed using the general linear model procedure in the SAS statistical software package (SAS Institute Inc., Cary, NC, USA). Park et al. investigated the genetic architecture of these traits and reported that the traits had moderate and high heritability estimates [22]. The previous studies on the association between genotypes and BVs considered BVs estimates of all additive genetic effects without the other environmental effects—and no environmental factor was included in the model [23,24]. Therefore, we followed their approaches in the association analysis for MAE SNPs. The statistical model was as follows: *y_ij_* = *μ* + *G_i_* + *e_ij_*, where *y_ij_* is the breeding value, *μ* is the general mean, *G_i_* is the fixed effect of genotype *i*, and *e_ij_* is the random error. Statistical significance was determined by *p* < 0.05. Association results are shown as the least-square means and standard error.

We identify the enriched gene ontology (GO) terms or the Kyoto encyclopedia of genes and genomes (KEGG) pathways for MAE genes using the database for annotation, visualization, and integrated discovery (DAVID) bioinformatics resources [25]. The results with *p* < 0.1 were considered significant enrichments. The networks based on association results and enriched terms were visualized in Cytoscape [26].

## 3. Results

### 3.1. Identification of SNPs with MAE

To determine monoallelic expressions of SNPs with heterozygous genotypes in offspring, whole genome resequencing for blood samples from sire and dam and RNA-sequencing for six tissues from offspring were conducted. Descriptive statistics for the sequencing data are in Table S1. After trimming the raw reads from re-sequencing, an average of 488 million (M) clean reads per sample were obtained, of which on average 94.2% were aligned with the bovine reference genome sequences. RNA-seq libraries generated an average of 49 M clean reads from six tissues. On average, 92.4% of clean reads were mapped to the genome.

**Figure 1:**
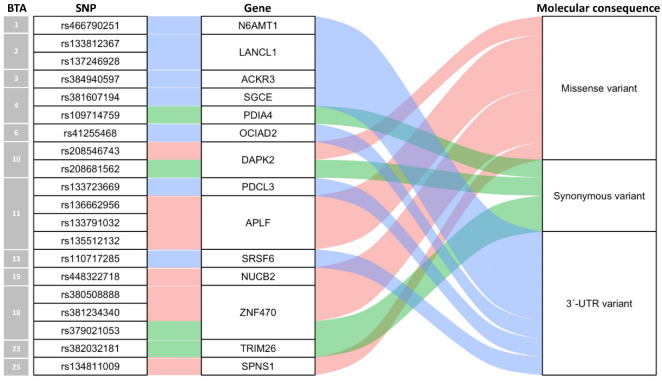
SNPs with monoallelic expressions in Hanwoo.

From variants calling for the parents’ resequencing data, a total of 5,898,127 SNPs were identified, of which 96,139 SNPs (1.63%) were assigned to gene coordinates. To obtain heterozygous exonic SNPs in offspring, we screened SNPs in which sire and dam were homozygous with opposite alleles. A total of 3801 SNPs remained, which were within 1569 annotated bovine genes. Using variant calling for all transcriptome data of six tissues from the offspring, the expressed alleles at these SNP loci were determined and used in the identification of allele-specific expression patterns and parental origin of the expressed alleles. Figure 1 shows 20 SNPs with MAE in at least 1 tissue of offspring in which 8 were missense variants, 4 were synonymous variants, and 8 were 3’-UTR variants. These SNPs were assigned to 14 genes located across 12 different chromosomes.

### 3.2. Tissue-Specific MAE Genes

**Figure 2:**
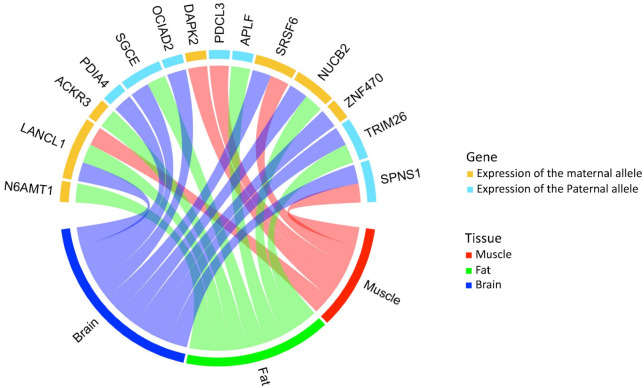
Tissue-specific monoallelic expression of 14 genes in muscle, fat, and brain tissues. The upper and lower hemispheres of a circle plot indicate genes and tissues, respectively. Yellow and light blue indicate the parental origin of the expressed alleles. The MAE status for each tissue is represented by red, green, and blue.

Figure 2 shows a tissue-specific MAE status of genes and the parental origin of their expressed allele. The number of genes in which maternal alleles were expressed and the number of genes in which paternal alleles were expressed were both seven. Only *LanC like 1* (*LANCL1*), whose maternal allele was expressed, showed MAEs across all three tissues, and the MAE status of the other genes varied depending on the tissue. There were two muscle specific MAE genes: *death associated protein kinase 2* (*DAPK2*) and *phosducin like 3* (*PDCL3*); three fat specific MAE genes: *N-6 adenine-specific DNA methyltransferase 1* (*N6AMT1*), *atypical chemokine receptor 3* (*ACKR3*), *aprataxin and PNKP like factor* (*APLF*); and three brain-specific MAE genes: *protein disulfide isomerase family A member 4* (*PDIA4*), *OCIA domain containing 2* (*OCIAD2*), and *ZNF470*. The remaining five genes showed MAEs in two types of tissues.

As shown in Table 1, we compared the identified genes with databases that provide the gene status of autosomal monoallelic expression or imprinting in multiple species, such as dbMAE (https://mae.hms.harvard.edu, accessed on 1 October 2021) and Geneimprint (http://www.geneimprint.com/, accessed on 1 October 2021). Ten of the 14 MAE genes in this study were overlapped with MAEs in mice and/or humans in dbMAE, supporting the adequacy of the MAE screening approach in this study. In addition, *sarcoglycan epsilon* (*SGCE*) was reported as an imprinted gene with a paternal allele expression in cattle, mice, humans, and pigs, corresponding to its MAE status in this study. The comparison results indicated that four genes, *PDCL3*, *N6AMT1*, *TRIM26*, and *ZNF470*, were novel MAE genes in cattle.

### 3.3. Association Anlaysis with BVs and Functional Annotations of MAE Genes

Table 2 shows the results of association analysis using the KPN population. The BVs for five major selection traits for Hanwoo, including WT12, CWT, LEA, BFT, and MAR, were obtained from the Hanwoo Improvement Center. In the KPN population, 16 of 20 MAE SNPs were polymorphic, covering 11 MAE genes. The frequencies of homozygotes of minor alleles were very low in most SNPs except for *APLF*, which had three completely linked SNPs. Therefore, the genotypes with very low frequencies (<10%) at each locus were excluded from statistical analysis. A total of six genes showed significant associations with at least one trait (Table 2). The SNPs of *N6AMT1*, *PDLA4*, and *PDCL3* (rs466790251, rs109714759, and rs109714759, respectively) were significantly associated with three traits: WT12, CWT, and LEA (*p* < 0.05). The *ACKR3* SNP (rs384940597) influenced breeding values of WT21 (*p* < 0.001) and CWT (*p* = 0.018), and the *APLF* SNPs showed association with only CWT (*p* < 0.049). Only *SNPNS1* influenced MAR (*p* = 0.027).

The functional annotation for MAE genes revealed that biological processes, including cell death, positive regulation of epithelial cell proliferation, regulation of intrinsic apoptotic signaling pathway, regulation of immune system process, and angiogenesis were enriched for MAE genes despite a limited number of identified MAE genes. There was no enriched KEGG pathway.

**Figure 3:**
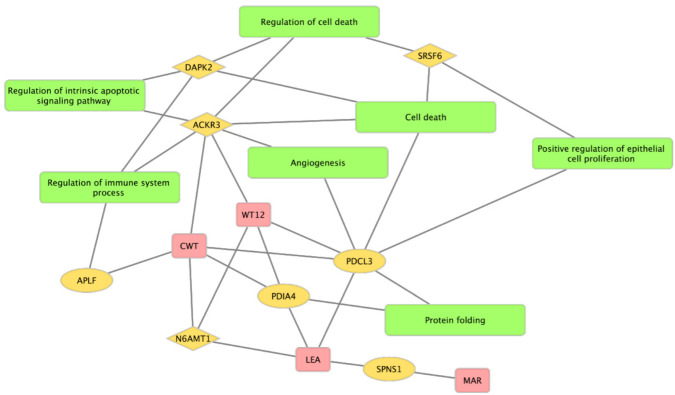
The network based on the enriched biological processes and associated traits for MAE genes. The node in yellow indicates genes whose shapes of diamonds and circles represent their expressed alleles maternally or paternally, respectively. The rectangles in green and red indicate the enriched biological processes and the associated traits, respectively.

Figure 3 shows the relationship network among MAE genes, associated traits, and biological functions. *ACKR3*, whose maternal allele was expressed, was involved in multiple functions, such as cell death, angiogenesis, immune system, and apoptotic signaling, and had an impact on weight gain-related traits, such as WT and CWT. In addition, *PDCL3* SNP was associated with three traits (WT12, CWT, and LEA) and was associated with biological processes such as cell death, angiogenesis, epithelial cell proliferation, and protein folding.

## 4. Discussion

The objectives of this study were to identify MAE genes and to investigate their biological features and relationships with BVs of economically essential traits in Hanwoo. We performed resequencing and RNA-sequencing in three adult animals in the same family to apply a SNP-based MAE evaluation approach as described by Lim et al. [16]. To overcome the limited number of animals, only the SNPs in which the sire and dam had different homozygous genotypes with sufficient depth of reads at the loci were determined as heterozygous SNPs for offspring. Based on this, we further specified parental origins of expressed alleles from the transcriptome data of various tissues from offspring, providing the basis for a possible imprinting study. 

A total of 1569 genes were evaluated, of which 14 were assessed for MAE. Most of the identified genes have been reported to show MAE patterns in humans and mice as well, based on dbMAE (https://mae.hms.harvard.edu/, accessed on 1 October 2021), while the MAE of four genes (*PDCL3*, *N6AMT1*, *TRIM26*, and *ZNF470*) were newly found in this study. The database of Geneimprint (http://www.geneimprint.com/, accessed on 1 October 2021) provides information on the paternal imprinting status of *SGCE* across multiple species, which is supported by previous reports in humans [27,28], mice [29], and pigs [30]. The paternal allele of *SGCE* was expressed in adult mouse tissues (brain, heart, and kidney) [31] and human peripheral blood leukocytes [27]. *SGCE* encodes epsilon-sarcoglycan and is associated with embryonic lethality and myoclonus–dystonia syndrome. In addition, *SGCE* is broadly expressed in embryos, and bovine *SGCE* expression peaked during an early embryonic stage [32]. We also identified paternal expression of bovine *SGCE* in adult fat and brain tissues corresponding to a previous report on the adult human brain [33]. Therefore, we hypothesize that the imprinting status of *SGCE* is maintained over an organism’s lifetime. However, significant effects of the *SGCE* SNP (rs381607194) on the BVs were not found in the KPN population. It is possible that individuals with deficiencies, such as myoclonus–dystonia syndrome, are not selected as KPNs. However, the minor allele frequency was sizable (0.28); therefore, further validation of *SGCE* genomic variants in the commercial population is required.

In this study, SNPs with MAEs were classified as missense variants, synonymous variants, and 3′UTR variants based on their molecular consequences. Most of the significant associations with BVs in KPNs were found in missense variants and 3′UTR variants, with the exception of the *PDIA4* SNP. The missense variants that alter the encoded amino acids could lead to changes in the structures and/or functions of proteins and previous studies have reported its impact on not only production traits, such as fat content, mortality, and milk yield, but also gene expression levels in cattle [34,35,36]. In addition, genomic variation in the 3′UTR of genes may affect the binding affinity of microRNAs, and its impact on phenotypic variations have been investigated in cattle [37,38,39].

The MAE patterns were identified across the genome and showed a tissue-specific manner in Hanwoo, corresponding to the previous report [16]. Similar trends in MAEs that were widespread on autosomes and varied between cell types have been reported in mice [40] and humans [41]. Xu et al. [42] reported the relationships between tissue-specific MAE and DNA methylation in bovine *AXL*. In general, methylation in the promoter regions of one of the two alleles of the gene often leads to allele silencing (reviewed by [43]). In this study, only *LANCL1* showed an MAE pattern across all tissues with an expression of the maternal allele. *LanCL1* is homologous with a prokaryotic enzyme related to antimicrobial peptide synthesis, whose expression in the brain has previously been reported in mice [44] and cattle [45]. In our population, however, there was no relationship with the traits utilized in the study. One of the muscle-specific MAE genes, *PDCL3,* encodes a chaperone protein, which is involved in angiogenesis by interacting with vascular endothelial growth factor receptor 2 [46]. The relationships between polymorphism in exon of *PDCL3* and economic traits, such as feed conversion ratio in broiler chickens, have been previously reported [47]. As shown in the functional network in Figure 3, *PDCL3* was connected to multiple biological processes, such as angiogenesis, cell death, epithelial cell proliferation, and protein folding, and had significant associations with growth (WT12) and lean meat production ability (CWT and LEA). Interestingly, all genes (*N6AMT1*, *APLF,* and *ACKR3*) that had fat-specific MAEs showed significant associations with at least one trait. *N6AMT1* is involved in the methylation of release factor 1 during termination of translation and is considered to be essential for embryo viability [48]. In addition, polymorphisms of *N6AMT1* were significantly related to arsenic metabolism in humans [49]. *APLF*, which has a GO term of regulation of immune system processes, was known to be involved in the cellular response to DNA strand breaks in human cells [50]. The network revealed that *ACKR3* was involved in various terms related to the immune system, apoptotic signaling, cell death, and angiogenesis, supporting its effects on CWT and WT12. Previous studies have reported that *ACKR3*, known as C-X-C chemokine receptor 7, is related to the function of myeloid lineage [51], and autoimmune diseases [52]. Additionally, *ACKR3* is characterized by the expression of maternal alleles, implying that it is a possible target for cows to enhance gene combination effects.

The parental differentiation of the trait-associated genes leads to changes in genetic variance and genetic gain in the population compared to non-imprinted genes or SNPs. The use of imprinted genetic markers could be designed more effectively in the breeding program. Among eight SNPs, two SNPs, *PDIA4* and *SPNS1*, with the expression of paternal alleles, are included in the bovine SNP chips and thus they may be more easily integrated into genomic selection for bulls. Additionally, the MAE genes with expression of maternal alleles such as *N6AMT1* and *ACKR3* could be the targets of marker-assisted selection for cows.

## 5. Conclusions

We identified 14 MAE genes in various adult tissues in Hanwoo and investigated their impacts on WT12, CWT, LEA, BFT, and MAR. Based on biological features and the relationships with genetic potentials for important economic traits, *PDIA4*, *SPNS1*, *N6AMT1*, and *ACKR3* were considered selective targets for breeding programs in Hanwoo. 

## Figures and Tables

**Table 1:** Genes with monoallelic expressions in muscle, fat, and brain tissues of Hanwoo.

Gene			Database	
Ensembl ID	Symbol	Expressed Allele	dbMAE ^1^	Geneimprint ^2^
ENSBTAG00000015066	*LANCL1*	Maternal	M	-
ENSBTAG00000011820	*DAPK2*	Maternal	M and H	-
ENSBTAG00000009962	*PDCL3*	Paternal	-	-
ENSBTAG00000008527	*SRSF6*	Maternal	M	-
ENSBTAG00000014653	*SPNS1*	Paternal	M and H	-
ENSBTAG00000001412	*N6AMT1*	Maternal	-	-
ENSBTAG00000018424	*ACKR3*	Maternal	M	-
ENSBTAG00000021282	*SGCE*	Paternal	M and H	C, M, H, and P
ENSBTAG00000018401	*APLF*	Paternal	M and H	-
ENSBTAG00000017468	*NUCB2*	Maternal	M	-
ENSBTAG00000035744	*TRIM26*	Paternal	-	-
ENSBTAG00000017143	*PDIA4*	Paternal	M	-
ENSBTAG00000001839	*OCIAD2*	Paternal	M	-
ENSBTAG00000046101	*ZNF470*	Maternal	-	-

Abbreviations: BTA, Bos taurus chromosome; C, cattle; H, human; M, mouse; P, pig. ^1^ dbMAE, https://mae.hms.harvard.edu, accessed on 1 October 2021. ^2^ Geneimprint, http://www.geneimprint.com/, accessed on 1 October 2021.

**Table 2:** Effects of MAE SNPs on breeding values of traits measured in Hanwoo.

Gene	SNP ID	Allele	Genotype Frequency ^1^			Associated Traits (*p*-Value)
			AA	AB	BB	
*N6AMT1*	rs466790251	G/A	124 (0.61)	71 (0.35)	8 (0.04) ^2^	WT12 (*p* = 0.005) CWT (*p* < 0.001) LEA (*p* < 0.001)
*ACKR3*	rs384940597	T/C	122 (0.60)	73 (0.36)	8 (0.04) ^2^	WT12 (*p* < 0.001) CWT (*p* = 0.018)
*PDIA4*	rs109714759	C/T	157 (0.77)	45 (0.22)	1 (0.00) ^2^	WT12 (*p* < 0.001) CWT (*p* = 0.003) LEA (*p* = 0.005)
*PDCL3*	rs133723669	T/C	124 (0.61)	71 (0.35)	8 (0.04) ^2^	WT12 (*p* = 0.030) CWT (*p* = 0.042) LEA (*p* < 0.001)
*APLF*	rs136662956 rs133791032 rs135512132	A/G G/A C/T	80 (0.39)	97 (0.48)	26 (0.13)	CWT (*p* = 0.049)
*SPNS1*	rs134811009	A/G	182 (0.90)	21 (0.10)	0 (0.00) ^2^	LEA (*p* = 0.004) MAR (*p* = 0.027)

Abbreviations: WT12, breeding value for weight at 12 months of age; CWT, breeding value for carcass weight; LEA, breeding value for loin eye area; MAR, breeding value for marbling. ^1^ The genotypes were represented by the combination of alleles A and B, of which B indicated the minor allele. ^2^ Genotype that was excluded from the association analysis.

## Data Availability

The RNA sequencing data have been uploaded to the NCBI SRA database with the accession numbers (SAMN23489129, SAMN23489130, SAMN23489131, SAMN23489132, SAMN23489133, and SAMN23489134). The resequencing data are not publicly available, but they can be made available by the corresponding author upon reasonable request.

## Supplementary Materials

The following are available online at https://www.mdpi.com/article/10.3390/ani12010084/s1, Table S1: Summary statistics for resequencing and RNA-sequencing data.
