# Supplementary material for: Identification of Monoallelically Expressed Genes Associated with Economic Traits in Hanwoo (Korean Native Cattle)
Source: Animals (Basel). 2021 Dec 31;12(1):84. doi: 10.3390/ani12010084 (PMC8749587; doi:10.3390/ani12010084)
Supplement: Supplementary file 1 [file animals-12-00084-s001.zip › animals-1433375-SI.pdf]

Table S1. Summary statistics for Re-sequencing and RNA-sequencing data.

Sequencing	ID	Sample	Read 1	Read 2	Total reads	Mapped reads	Mapped rate
Re-seq	Sire	Blood	265,049,733	265,049,733	530,099,466	494,051,940	93.20%
	Dam	Blood	253,291,721	253,291,721	506,583,442	482,508,144	95.25%
RNA-seq	Offspring	Loin muscle	28,606,852	28,606,852	57,213,704	52,928,967	92.51%
		Femoral muscle	23,248,921	23,248,921	46,497,842	43,816,858	94.23%
		Backfat	25,069,553	25,069,553	50,139,106	46,475,064	92.69%
		Abdominal fat	22,528,446	22,528,446	45,056,892	39,677,151	88.06%
		Pituitary gland	24,057,648	24,057,648	48,115,296	45,215,265	93.97%
		Hypothalamus	22,693,187	22,693,187	45,386,374	42,282,881	93.16%
